# Development and validation of a risk score to predict mortality during TB treatment in patients with TB-diabetes comorbidity

**DOI:** 10.1186/s12879-018-3632-5

**Published:** 2019-01-05

**Authors:** Duc T. Nguyen, Edward A. Graviss

**Affiliations:** Department of Pathology and Genomic Medicine, Houston Methodist Research Institute, Mail Station: R6-414, 6670 Bertner Ave, Houston, TX 77030 USA

**Keywords:** Tuberculosis, Diabetes, Risk score, Mortality, TB-diabetes, TB-DM

## Abstract

**Background:**

Making an accurate prognosis for mortality during tuberculosis (TB) treatment in TB-diabetes (TB-DM) comorbid patients remains a challenge for health professionals, especially in low TB prevalent populations, due to the lack of a standardized prognostic model.

**Methods:**

Using de-identified data from TB-DM patients from Texas, who received TB treatment had a treatment outcome of completed treatment or died before completion, reported to the National TB Surveillance System from January 2010–December 2016, we developed and internally validated a mortality scoring system, based on the regression coefficients.

**Results:**

Of 1227 included TB-DM patients, 112 (9.1%) died during treatment. The score used nine characteristics routinely collected by most TB programs. Patients were divided into three groups based on their score: low-risk (< 12 points), medium-risk (12–21 points) and high-risk (≥22 points). The model had good performance (with an area under the receiver operating characteristic (ROC) curve of 0.83 in development and 0.82 in validation), and good calibration. A practical mobile calculator app was also created (https://oaa.app.link/Isqia5rN6K).

**Conclusion:**

Using demographic and clinical characteristics which are available from most TB programs at the patient’s initial visits, our simple scoring system had good performance and may be a practical clinical tool for TB health professionals in identifying TB-DM comorbid patients with a high mortality risk.

**Electronic supplementary material:**

The online version of this article (10.1186/s12879-018-3632-5) contains supplementary material, which is available to authorized users.

## Background

The effect of diabetes mellitus (DM) on the development and poor outcome of tuberculosis (TB) disease has been recognized for over a century [1]. While diabetes ranked 7th among the leading causes of death in 2015, TB has been recognized as a leading cause of the mortality due to an infectious disease [2, 3]. With the global increase of obesity and type 2 diabetes, the combination of diabetes and tuberculosis (TB-DM) has posed an imminent public health threat and a challenge to TB control programs worldwide [4]. In the United State (US), the prevalence of diabetes has consistently increased from 0.93% in 1958 to 7.40% in 2015 with an estimate of 30.3 million people of all ages (9.4% of the US population) living with diabetes [5, 6]. This increasing trend of diabetes morbidity in the US is concerning especially in US states (such as Texas) where both the TB and the DM prevalence are higher than the national average [7, 8]. Given that TB-DM comorbid patients may have a mortality of 2–5 times higher than that of non-diabetic TB patients [9, 10], more effective management strategies including the development of predictive models for TB mortality are urgently needed.

There are a growing number of prognostic models developed to predict mortality in patients with TB disease. Many characteristics such as older age, HIV co-infection, diabetes, alcohol abuse, malnutrition, hypoxemic respiratory failure, etc. have been identified as the risk factors for poor outcomes in TB patients [11–15]. However, these models were not specifically developed for patients with TB-DM comorbidity and used hospital-based data with variables that are not routinely collected by TB control programs. The populations of these models either did not include diabetic patients [11, 12] or only included a small number of TB-DM patients [13–15].

The lack of a standardized prognostic system specifically developed for TB-DM patients poses a challenge for health care providers attempting to predict the risk of mortality during TB treatment in this high-risk group of patients. The present study aimed to develop and internally validate a prognostic scoring system using surveillance data with covariates which are routinely collected by most TB control program and available at the patient’s initial visits for TB evaluation. This simple scoring system would be a practical tool helping quickly identify TB-DM patients having a high risk of death during TB treatment.

## Methods

This retrospective cohort study used the de-identified data of all confirmed TB patients from the state of Texas reported to the Centers for Disease Control (CDC)‘s National TB Surveillance System (NTSS) between January 2010 through December 2016 (both genotyped and non-genotyped), who satisfied the following inclusion criteria: (1) met the clinical case definition or was laboratory confirmed based on the CDC definition for a TB case [16]; (2) received TB treatment and had a documented outcome of either “completed” or “died”. Patients having treatment outcomes other than “completed” or “died” (such as “adverse”, “lost”, “moved”, “other”, “refused”, or “unknown”) were excluded from the analyses.

Logistic regression modeling was used to determine prognostic factors associated with patient mortality. Variables with a *p*-value < 0.2 in the univariate analysis or considered as clinically significant were evaluated further in the multiple logistic regression. The variable section for the multiple logistic regression model was conducted according the Bayesian Modeling Averaging (BMA) method [17, 18]. As our goal was to develop a model that could be used in the patient’s initial visit when the *Mtb* biological confirmation is still not available, *Mtb* culture and genotype-related variables were not evaluated in the multivariable modeling. Model discrimination was determined by the area under the Receiver Operating Characteristic (ROC) curve (AUC). The best model was chosen based on the smallest Bayesian information criterion and highest AUC. The model’s good calibration was determined by a non-significant Hosmer-Lemeshow’s goodness of fit test.

Significant risk factors were assigned weighted-points that were proportional to their β regression coefficient values. A prognostic score was calculated for each individual patient in the cohort. The methodology of categorizing risk groups has been described elsewhere [19, 20]. Briefly, the patients were categorized in three distinct groups of mortality risk: low (< 10% mortality), medium (10–20% mortality), and high risk (> 20% mortality). Internal validation was conducted using the bootstrap resampling method with 2000 replications. Model calibration was evaluated by the Hosmer-Lemeshow goodness-of-fit test. A non-significant *p*-value of the Hosmer-Lemeshow goodness-of-fit test indicates the model has a good calibration (predictive accuracy). The comparison of the AUC between models was conducted using the chi-square test. All analyses were performed with Stata MP14.2 (StataCorp LLC, College Station, TX). A *p* value < 0.05 was considered statistically significant.

## Results

**Fig. 1:**
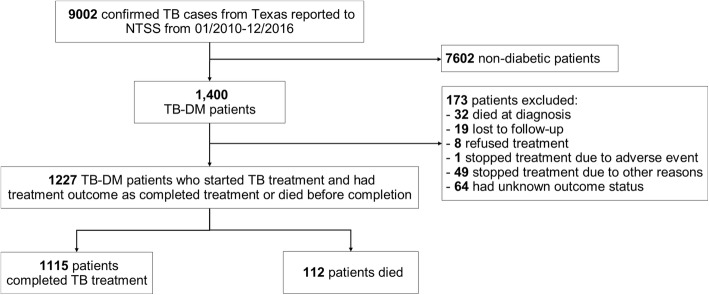
Flowchart of the study population. NTSS: National Tuberculosis Surveillance System

Between January 2010 and December 2016, 1400 TB-DM patients in Texas were reported in the National TB Surveillance System database. After excluding 173 patients who had an outcome other than “completed” or “died”, 1227 TB-DM patients, who started the TB treatment and had a treatment outcome of completed treatment or died before completion, were included in the analysis, of whom 1115 completed TB treatment and 112 died (Fig. 1). Except for injecting-drug user (IDU) (*p* = 0.01), no other difference was found between the patients who were included in the analysis and those who were excluded (Additional file 1: Table S1).

The crude and adjusted associations between characteristics and mortality are presented in Table 1. Nine variables (age ≥ 65 years, being US-born, being homeless, IDU, having chronic kidney disease, TB meningitis, miliary TB, positive acid-fast bacilli (AFB) smear, and positive HIV status) were significant in the multiple logistic regression model and were included in the risk score development. The weighted points of risk factors were calculated using the linear transformation of the corresponding β coefficient (Table 2) [21]. A risk score was calculated for individual patients using the following formula:

$$ {\displaystyle \begin{array}{c}\mathrm{Risk}\ \mathrm{score}={16}^{\ast}\left[\mathrm{Age}\ge 65\right]+{5}^{\ast}\left[\mathrm{US}-\mathrm{born}\right]+{11}^{\ast}\left[\mathrm{Homeless}\right]+{20}^{\ast}\left[\mathrm{IDU}\right]+{20}^{\ast}\left[\mathrm{Chronic}\ \mathrm{kidney}\ \mathrm{failure}\right]\\ {}+{20}^{\ast}\left[\mathrm{TB}\ \mathrm{meningitis}\right]+{13}^{\ast}\left[\mathrm{Miliary}\ \mathrm{TB}\right]+{6}^{\ast}\left[\mathrm{AFB}\left(+\right)\ \mathrm{smear}\right]+{24}^{\ast}\left[\mathrm{Positive}\ \mathrm{HIV}\right].\end{array}} $$

**Table 1:** Characteristics associated with mortality during tuberculosis treatment

	Total	Completed	Deceased	Unadjusted OR	Adjusted OR	Adjusted p
	(*N* = 1227)	(*n* = 1115)	(*n* = 112)	(95% CI)	(95% CI)	
Age ≥ 65 (years)	322 (26.2)	260 (23.3)	62 (55.4)	4.08 (2.74, 6.07)	4.85 (3.00, 7.85)	< 0.001
Male gender	790 (64.4)	712 (63.9)	78 (69.6)	1.30 (0.85, 1.98)		
Race/Ethnicity						
White	90 (7.3)	78 (7.0)	12 (10.7)	1.80 (0.93, 3.48)		
Black	136 (11.1)	119 (10.7)	17 (15.2)	1.67 (0.95, 2.95)		
Hispanic	827 (67.4)	762 (68.3)	65 (58.0)	(reference		
Asian	164 (13.4)	147 (13.2)	17 (15.2)	1.36 (0.77, 2.38)		
Other	10 (0.8)	9 (0.8)	1 (0.9)	1.30 (0.16, 10.44)		
US-born	450 (40.0)	396 (38.7)	54 (51.9)	1.70 (1.14, 2.50)	1.64 (1.03, 2.62)	0.04
Homeless	41 (3.3)	33 (3.0)	8 (7.1)	2.52 (1.14, 5.60)	2.88 (1.05, 7.88)	0.04
Inmate in a correctional institution	35 (3.3)	33 (3.4)	2 (2.1)	0.61 (0.14, 2.58)		
Resident of long-term care facility	21 (1.7)	14 (1.3)	7 (6.3)	5.24 (2.07, 13.28)	1.50 (0.49, 4.58)	0.47
IDU addicts	15 (1.2)	10 (0.9)	5 (4.5)	5.16 (1.73, 15.38)	7.25 (1.64, 31.98)	0.01
Non-IDU addicts	73 (5.9)	64 (5.7)	9 (8.0)	1.43 (0.69, 2.97)		
Excessive alcohol consumption	182 (14.8)	174 (15.6)	8 (7.1)	0.42 (0.20, 0.87)	0.46 (0.20, 1.07)	0.07
Organ transplant receiver	8 (0.7)	7 (0.6)	1 (0.9)	1.43 (0.17, 11.70)		
Chronic kidney failure	60 (4.9)	38 (3.4)	22 (19.6)	6.93 (3.93, 12.22)	7.00 (3.59, 13.62)	< 0.001
Immunosuppression (medical condition or medication)	35 (2.9)	30 (2.7)	5 (4.5)	1.69 (0.64, 4.45)		
Pulmonary TB	1110 (90.5)	1011 (90.7)	99 (88.4)	0.78 (0.42, 1.45)		
TB meningitis	10 (0.8)	7 (0.6)	3 (2.7)	4.36 (1.11, 17.09)	7.25 (1.54, 34.08)	0.01
Miliary TB	33 (2.7)	25 (2.3)	8 (7.1)	3.32 (1.46, 7.55)	3.79 (1.41, 10.21)	0.01
TB-CXR	1084 (88.3)	983 (88.2)	101 (90.2)	1.23 (0.64, 2.36)		
Cavitation on CXR	509 (47.0)	479 (48.7)	30 (29.7)	0.44 (0.28, 0.69)	0.69 (0.41, 1.15)	0.15
AFB smear (+)	684 (61.4)	636 (61.6)	48 (58.5)	0.88 (0.56, 1.39)	1.74 (1.01, 2.98)	0.04
Culture (+)	863 (70.3)	799 (71.7)	64 (57.1)	2.52 (1.73, 3.68)		
TB case verified by positive culture, NAA, or AFB smear	1066 (86.9)	964 (86.5)	102 (91.1)	1.60 (0.82, 3.13)		
HIV status (+)	23 (1.9)	15 (1.3)	8 (7.1)	7.40 (3.04, 18.03)	11.07 (3.66, 33.46)	< 0.001
MDR-TB	10 (0.8)	8 (0.7)	2 (1.8)	2.42 (0.51, 11.54)		
*Mtb* East Asian lineage	128 (13.2)	114 (13.0)	14 (15.2)	1.20 (0.66, 2.20)		

*CXR* chest radiograph, *TB-CXR* abnormalities on CXR consistent with tuberculosis, *MDR-TB* Multi-drug resistant TB, *NAA* Nucleic Acid Amplification; Excessive alcohol consumption, excessive alcohol consumption in the past 12 months; *Mtb, Mycobacterium tuberculosis*

**Table 2:** Weighted score assignment

Variable	β coefficient	Adjusted OR (95% CI)	*P* Value	Weighted Points
Age ≥ 65 (years)	1.58	4.85 (3.00, 7.85)	< 0.001	16
US-born	0.49	1.64 (1.03, 2.62)	0.04	5
Homeless	1.06	2.88 (1.05, 7.88)	0.04	11
IDU	1.98	7.25 (1.64, 31.98)	0.01	20
Chronic kidney failure	1.95	7.00 (3.59, 13.62)	< 0.001	20
TB meningitis	1.98	7.25 (1.54, 34.08)	0.01	20
Miliary TB	1.33	3.79 (1.41, 10.21)	0.01	13
Positive AFB smear	0.55	1.74 (1.01, 2.98)	0.04	6
Positive HIV	2.40	11.07 (3.66, 33.46)	< 0.001	24

Weighted points of a risk factor were calculated using a linear transformation of the corresponding β coefficient [divided by the smallest β coefficient (0.49, US-born), multiplied by a constant (5), and rounded to the nearest integer] [21]

Risk score = 16*[Age ≥ 65] + 5*[US-born] + 11*[Homeless] + 20*[IDU] + 20*[Chronic kidney failure] + 20*[TB meningitis] + 13*[Miliary TB] + 6*[AFB-positive smear] + 24*[Positive HIV]

*IDU* injecting-drug user, *AFB* acid-fast bacilli

**Fig. 2:**
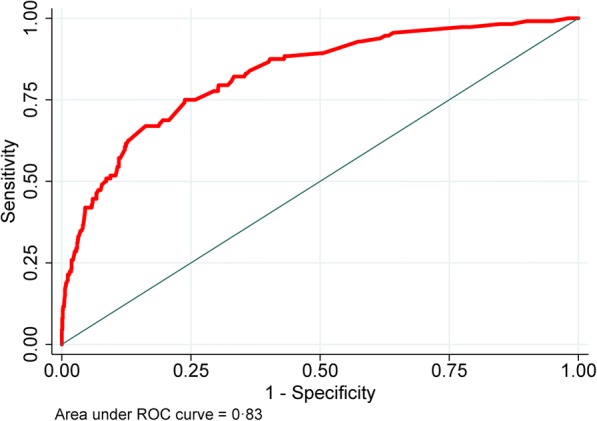
Area under the ROC curve, final model. ROC: Receiver Operating Characteristic curve

There were 776 (63.7%) low-risk, 233 (19.2%) medium risk and 208 (17.1%) high-risk patients with the mortality by risk group of 3.1, 12.9 and 27.9%, respectively. The final model had good discrimination in both development (AUC = 0.83 95% CI 0.79, 0.87) and bootstrap validation (AUC = 0.82 95% CI 0.78, 0.87) (Table 3, Fig. 2). The model also had a good calibration with a non-significant Hosmer-Lemeshow chi-square of 4.54 (*p* = 0.81) and a small Brier score of 0.07 (Table 3). Patients in the medium- and high-risk groups had more than a four- and twelve-fold increased odds of mortality compared with patients in the low-risk group (Table 4). We also compared the performance of the current TB-DM specific model and that of our previously-published mortality predictive model, which included all confirmed TB patients who started TB treatment [19]. In TB-DM patients who were included in this study, the TB-DM specific model had a significantly higher discrimination power than that of its predecessor [AUC 0.83 (95% CI 0.79, 0.88) versus 0.76 (0.71, 0.82), *p* < 0.001] (data not shown).

**Table 3:** Prognostic score performance in patients with complete data for multivariate model (*N* = 1113)

Risk group	n (%)	Score, mean (±SD)	Mortality % (n)	*P* Value*
Low-risk group (< 12 points)	776 (63.7%)	4.8 (±3.8)	3.1% (24)	< 0.001
Medium-risk group (12–21 points)	233 (19.2%)	17.3 (±2.3)	12.9% (30)	
High-risk group (≥22 points)	208 (17.1%)	27.7 (±7.3)	27.9% (58)	
All patients with complete data (*N* = 1217)	1217 (100%)	11.1 (±10.0)	9.2% (112)	
Discrimination assessment				
AUC (95% CI), final model in development	0.83 (0.79, 0.87)			
AUC (95% CI), final model in bootstrap validation	0.82 (0.78, 0.87)			
Calibration assessment				
Hosmer-Lemeshow test	Chi-square = 4.54, *p* = 0.81			
Brier score	0.07			

Abbreviation: *SD* standard deviation, *AUC* Receiver Operating Characteristic (ROC) curve

Score range: [0–68]; Comparisons of mortality between risk groups were conducted using Chi-square test; *Overall *p*-value. A *p* < 0.01 was also found for all pairwise comparisons among groups (i.e. low-risk vs. medium-risk, low-risk vs. high-risk and medium-risk vs. high-risk groups)

Brier score: ranged from 0 to 1, the lower the Brier score is, the better the model is calibrated; Hosmer-Lemeshow test: non-significant *p*-value indicates the model is well calibrated

**Table 4:** Odds ratios for death, by risk group

Risk group	OR (95% CI)	*P* Value
Low-risk group (< 12 points)	(reference)	
Medium-risk group (12–21 points)	4.63 (2.65, 8.10)	< 0.001
High-risk group (≥22 points)	12.12 (7.30, 20.12)	< 0.001

The predicted probability of death during TB treatment can be calculated based on the intercept value (− 4.004594) of the final model and corresponding β coefficients of the variables included in the risk score, the predicted probability of death during TB treatment can be calculated from the following formula:

$$ {\displaystyle \begin{array}{c}\mathrm{Probability}\ \mathrm{for}\ \mathrm{death}=\exp \Big(-4.004594+{1.579789}^{\ast}\left[\mathrm{Age}\ge 65\right]+{0.4946987}^{\ast}\left[\mathrm{US}-\mathrm{born}\right]\\ {}+{1.05767}^{\ast}\left[\mathrm{Homeless}\right]+{1.980345}^{\ast}\left[\mathrm{IDU}\right]+{1.945451}^{\ast}\left[\mathrm{Chronic}\ \mathrm{kidney}\ \mathrm{failure}\right]+{1.981255}^{\ast}\left[\mathrm{TB}\ \mathrm{meningitis}\right]\\ {}+{1.332084}^{\ast}\left[\mathrm{Miliary}\ \mathrm{TB}\right]+{0.5537461}^{\ast}\left[\mathrm{AFB}-\mathrm{positive}\ \mathrm{smear}\right]+{2.404202}^{\ast}\left[\mathrm{Positive}\ \mathrm{HIV}\right]\Big).\end{array}} $$

### Online calculator

We have created a free online application for our risk score calculator, which can be downloaded from https://oaa.app.link/Isqia5rN6K and usable on both android and iOS mobile devices (registration for a free account of OpenAsApp is required to access the calculator). The calculator app provides a risk score (in points), risk group (low, medium or high), and probability of death (%) during treatment for an individual patient.

## Discussion

Using 7 years of TB surveillance data from the state of Texas, we have developed and internally validated a simple prognostic score to predict mortality during treatment of TB-DM patients using only nine variables, which are routinely collected by most TB control program at the patients’ initial visits for TB evaluation before the availability of the *Mtb* culture. Having good discrimination and calibration together with the availability of a calculator mobile app, the scoring system would be a practical tool for clinicians and public health professionals to quickly identify the TB-DM patients who have a high mortality risk without waiting for the biological confirmation. Our scoring system classifies patients into three distinctive risk groups, which would be helpful for health care workers in allocating the appropriate medical support and follow-up resources. While patients of the low-risk group can be managed according to the routine protocol, TB-DM patients in the high-risk group would need more aggressive medical support. Although many of our prognostic model’s components are unmodified characteristics, there are still multiple approaches that could be carried out to improve patient survival, especially patients in the high-risk group. Having better management of the plasma glucose level is among the important strategies to reduce the patient’s mortality as compared with TB patients with controlled DM. TB patients with uncontrolled DM have more than 4 times the odds for death and 2 times the odds of non-conversion of sputum cultures after 2 months of intensive treatment [22]. Education on the negative impacts of DM on TB patients as well as guidelines for changes in diet and physical activity should be provided to patients and their family so that they can be more compliant with the treatment and actively contribute to the glucose control improvement [23]. More aggressive nutritional support would be necessary for high-risk patients who are residents of long-term care facilities as these patients are also prone to other potential risk factors for TB mortality such as old age and under-nutritional condition [11]. Given that the combination antiretroviral therapy (cART) could reduce up to 68% TB-related deaths in TB/HIV co-infected patients [24], early initiation of cART could be considered in high-risk patients, who are also HIV positive, although cART is recommended to be started within 8 weeks of starting TB treatment if CD4+ level ≥ 50 cells/mm3 [25].

In our scoring system, a positive HIV status, having chronic kidney failure, TB meningitis, being IDU and age ≥ 65 years are strong predictors for mortality in TB-DM patients. These findings are consistent with current literature for TB patients in general [13, 26–29].

In a previous analysis using US multiple cause-of-death (MCOD) data from 1990 through 2006, Jung et al. observed that foreign-born patients had more than twice the TB-related death rate than that of US-born patients [30]. Meanwhile, our findings suggest that US-born TB-DM patients have more than twice the odds of death compared with foreign-born patients even after controlling for older age, homelessness, IDU, alcohol abuse and HIV infection. Our finding is consistent with the results reported by Magee et al. in a more recent population-based study using the state-wide surveillance data from 2009 to 2012 in Georgia, in which a significantly higher mortality in US-born patients was found in both non-diabetic and diabetic TB patients [31]. The possible reasons leading to a higher mortality in US-born than foreign-born TB patients have been discussed elsewhere [19]. Briefly, foreign-born suspected TB patients might promptly receive the diagnosis and aggressive management than the US-born patients as foreign-birth has been recognized as a strong risk factor for TB disease by the Texas TB program [32]. Early detection of TB cases among foreign-born persons which occurs during immigration screening, contact investigation and targeted testing may also play a role in relatively lowering the mortality risk in foreign-born patients compare to US-born patients. Lastly, the significantly higher proportion of US-born TB cases in Texas compared to the national average (41.3% versus 31.4% in 2016, *p* < 0.001) suggests that some US-born patients may not be timely diagnosed, especially those patients who do not have a recent history of travelling to high TB burden countries [33].

The impact of DM-TB on patient mortality was reported inconsistently among studies in different populations [31, 34]. In one of our previous studies using the surveillance data of all confirmed TB patients reported from the state of Texas between 2010 and 2016, the unadjusted association between mortality during treatment and diabetes was not significant (unadjusted odds ratio [OR] 1.04; 95% confidence interval [95% CI] 0.74, 1.47; *p* = 0.82) [19]. However, this unadjusted OR was obtained from only half of the study sample and might underestimate the impact of diabetes on the mortality. A more recent trend analysis using the entire data of the same population suggested that TB-DM patients had a higher mortality (10.3%) than non-DM patients (7.6%, *p* = 0.001) with nearly a 3-fold increase in the odds of overall death (adjusted OR 2.75; 95% CI 1.40, 5.39; *p* = 0.003) and death during TB treatment (adjusted OR 2.43; 95% CI 1.13, 5.23; *p* = 0.023). Additionally, while non-diabetic TB patients had a significant decrease in the mortality from 2010 to 2016, the mortality trend in TB-DM patients is unchanged overtime and consistently higher than that of non-diabetic patient (z = 3.05, *p* = 0.002) [35].

Although there is an increasing number of TB mortality risk scores being developed, we are not aware of a scoring system that is specific for the TB-DM population. In the TB mortality scoring systems presented by Horita (2013) and Pefura-Yone (2017), TB-DM patients were not included [11, 12]. In the prognostic models presented by Lui (2014), Nagai (2016), and Bastos (2016), only a small number of TB-DM patients were included in the study samples (ranged from *n* = 74 to *n* = 84) [13–15]. As these models used hospital-based data, many variables in these models such as respiratory failure requiring oxygen, serum albumin, activity of daily living, dehydration, hypoxemic respiratory failure, orientation disturbance, etc. are not routinely collected by TB programs. Using demographic and clinical characteristics in TB-DM patients, which are available for most TB program, our TB-DM mortality model is more practical and can be used in difference health care and public health settings. Although we have previously developed a mortality risk model including all patients staring TB treatment which has been shown to have a good overall diagnostic performance (AUC 0.82 in development and 0.80 in validation) in all presentations of TB in general [19], the model’s AUC decreases to 0.76 in TB-DM patients. When the variable selection was specifically calibrated for TB-DM patients, our new TB-DM specific predictive model provided a more accurate prognosis in the TB-DM population.

Our risk model has several notable limitations. First, the study excluded 233 patients who died at the diagnosis or had a treatment outcome of other than “died” or “completed”, which may be prone to potential misclassification bias. However, except for having a higher proportion of IDUs, the excluded patients had no other significant differences in the demographic and clinical characteristics compared with the included patients. Therefore, the misclassification bias, if any, would be minimal. Second, given our goal was using only the data routinely collected by the most TB programs, information regarding the diabetes treatment, lipid profiles and HbA1c were not evaluated in our model. Despite the lack of DM-specific variables, the model can still correctly discriminate the risk of mortality in most of the cases with an AUC of 0.83. Third, as our scoring system was developed in the US, external validation in settings of high TB burden would be necessary. Fourth, the use of surveillance data itself has some limitations. For example, certain self-reported data were obtained from interviewing TB patients which leads to a possibility that recall bias cannot be completely ruled out. Treatment failure or relapse were not well defined in the dataset. Treatment time and time to event data were not available and prevent us from performing more robust survival analyses. As our primary goal was to develop a predictive model for death during TB treatment, mortality prior to starting TB treatment has been excluded. Therefore, our findings may not reflect the overall mortality in TB-DM patients. Lastly, as Texas is one of the US states with a high TB burden, external validation in populations in different US states would also be needed.

Despite the limitations, there are several strengths making our prognostic score distinct: (1) using population-based surveillance data of the entire state of Texas during 7 years; (2) including exclusively TB-DM patients with a large sample size; (3) using demographic and clinical characteristics which are routinely collected by most TB programs from the initial patient visits, our model can be used to identify the TB-DM with high mortality risk before having the biological confirmation for *Mtb*; (4) having good discrimination in both development and bootstrap internal validation (AUC = 0.83 and 082, respectively) and good calibration; and (5) providing a simple scoring system with the available of a mobile app for easily calculating the predicted probability of death during TB treatment.

## Conclusions

Using demographic and clinical characteristics variables which are routinely collected by most TB programs from the initial patient visits, our simple scoring system can be used without waiting for the *Mtb* biological result and achieves good discrimination and calibration with the internal validation. With the free calculator app compatible with android and iOS mobile devices, the score would be a practical clinical tool for TB health professionals in identifying TB-DM comorbid patients who have high mortality risk so that appropriate approaches would be implemented to improve the patient outcomes.

## Additional file

Additional file 1: **Table S1.** Demographic and clinical characteristics of the study population compared with those excluded from the analyses. (DOCX 22 kb)

## Abbreviations

AFB: Acid-fast bacilli
AUC: Area under the Receiver Operating Characteristic curve
BMA: Bayesian modeling averaging
cART: Combination antiretroviral therapy
CDC: Centers for disease control
DM: Diabetes mellitus
IDU: Injecting-drug user
MCOD: Multiple cause-of-death
NTSS: National TB Surveillance System
ROC: Receiver operating characteristic
TB: Tuberculosis
TB-DM: Tuberculosis-diabetes
US: United State
