# Supplementary material for: Development and validation of a risk score to predict mortality during TB treatment in patients with TB-diabetes comorbidity
Source: BMC Infect Dis. 2019 Jan 5;19:10. doi: 10.1186/s12879-018-3632-5 (PMC6321653; doi:10.1186/s12879-018-3632-5)
Supplement: Supplementary file 1 — Table S1. Demographic and clinical characteristics of the study population compared with those excluded from the analyses. (DOCX 22 kb) [file 12879_2018_3632_MOESM1_ESM.docx]

**Additional file 1: Table S1. Demographic and clinical characteristics of the study population compared with those excluded from the analyses.**

|  | **All TB-DM patients*** | **Included** | **Excluded** | **p-value** |
| --- | --- | --- | --- | --- |
|  | **(N=1400)** | **(N=1227)** | **(N=173)** |  |
| Age (years) |  |  |  | 0.50 |
| 00-14 | 2 (0.1) | 2 (0.2) | 0 (0.0) |  |
| 15-24 | 17 (1.2) | 17 (1.4) | 0 (0.0) |  |
| 25-44 | 289 (20.6) | 255 (20.8) | 34 (19.7) |  |
| 45-64 | 719 (51.4) | 631 (51.4) | 88 (50.9) |  |
| ≥65 | 373 (26.6) | 322 (26.2) | 51 (29.5) |  |
| Gender |  |  |  | 0.49 |
| Female | 494 (35.3) | 437 (35.6) | 57 (32.9) |  |
| Male | 906 (64.7) | 790 (64.4) | 116 (67.1) |  |
| Race |  |  |  | 0.34 |
| White | 100 (7.1) | 90 (7.3) | 10 (5.8) |  |
| Black | 150 (10.7) | 136 (11.1) | 14 (8.1) |  |
| Hispanic | 947 (67.6) | 827 (67.4) | 120 (69.4) |  |
| Asian | 193 (13.8) | 164 (13.4) | 29 (16.8) |  |
| Other | 10 (0.7) | 10 (0.8) | 0 (0.0) |  |
| Hispanic |  |  |  | 0.61 |
| No | 1,300 (92.9) | 1,137 (92.7) | 163 (94.2) |  |
| Yes | 100 (7.1) | 90 (7.3) | 10 (5.8) |  |
| US-born |  |  |  | 0.72 |
| No | 889 (63.5) | 777 (63.3) | 112 (64.7) |  |
| Yes | 511 (36.5) | 450 (36.7) | 61 (35.3) |  |
| Homeless |  |  |  | 0.65 |
| No | 1,355 (96.8) | 1,186 (96.7) | 169 (97.7) |  |
| Yes | 45 (3.2) | 41 (3.3) | 4 (2.3) |  |
| Inmate in a correctional institution |  |  |  | 0.17 |
| No | 1,165 (96.4) | 1,029 (96.7) | 136 (94.4) |  |
| Yes | 43 (3.6) | 35 (3.3) | 8 (5.6) |  |
| Resident of long-term care facility |  |  |  | 0.54 |
| No | 1,375 (98.2) | 1,206 (98.3) | 169 (97.7) |  |
| Yes | 25 (1.8) | 21 (1.7) | 4 (2.3) |  |
| IDU |  |  |  | 0.01 |
| No | 1,378 (98.4) | 1,212 (98.8) | 166 (96.0) |  |
| Yes | 22 (1.6) | 15 (1.2) | 7 (4.0) |  |
| Non-IDU |  |  |  | 0.10 |
| No | 1,322 (94.4) | 1,154 (94.1) | 168 (97.1) |  |
| Yes | 78 (5.6) | 73 (5.9) | 5 (2.9) |  |
| Excessive alcohol use in the past 12 months |  |  |  | 0.82 |
| No or unknown | 1,190 (85.0) | 1,044 (85.1) | 146 (84.4) |  |
| Yes | 209 (14.9) | 182 (14.8) | 27 (15.6) |  |
| Unknown | 1 (0.1) | 1 (0.1) | 0 (0.0) |  |
| Chronic kidney failure |  |  |  | 0.88 |
| No | 1,332 (95.1) | 1,167 (95.1) | 165 (95.4) |  |
| Yes | 68 (4.9) | 60 (4.9) | 8 (4.6) |  |
| Pulmonary TB |  |  |  | 0.40 |
| No | 137 (9.8) | 117 (9.5) | 20 (11.6) |  |
| Yes | 1,263 (90.2) | 1,110 (90.5) | 153 (88.4) |  |
| TB meningitis |  |  |  | 0.08 |
| No | 1,386 (99.0) | 1,217 (99.2) | 169 (97.7) |  |
| Yes | 14 (1.0) | 10 (0.8) | 4 (2.3) |  |
| Miliary TB |  |  |  | 0.82 |
| No | 1,362 (97.3) | 1,194 (97.3) | 168 (97.1) |  |
| Yes | 38 (2.7) | 33 (2.7) | 5 (2.9) |  |
| TB-CXR |  |  |  | 0.45 |
| No | 172 (12.3) | 143 (11.7) | 29 (16.8) |  |
| Yes | 1,228 (87.7) | 1,084 (88.3) | 144 (83.2) |  |
| Cavitation on CXR |  |  |  | 0.18 |
| No | 660 (53.7) | 575 (53.0) | 85 (59.0) |  |
| Yes | 568 (46.3) | 509 (47.0) | 59 (41.0) |  |
| AFB smear |  |  |  | 0.66 |
| Negative | 482 (34.4) | 430 (35.0) | 52 (30.1) |  |
| Positive | 760 (54.3) | 684 (55.7) | 76 (43.9) |  |
| Not done/Unknown | 158 (11.3) | 113 (9.2) | 45 (26.0) |  |
| Culture |  |  |  | 0.79 |
| Negative | 277 (19.8) | 248 (20.2) | 29 (16.8) |  |
| Positive | 958 (68.4) | 863 (70.3) | 95 (54.9) |  |
| Not done/Unknown | 165 (11.8) | 116 (9.5) | 49 (28.3) |  |
| TB case verified by |  |  |  | 0.95 |
| Clinical case | 184 (13.1) | 161 (13.1) | 23 (13.3) |  |
| Positive culture, NAA, or AFB smear | 1,216 (86.9) | 1,066 (86.9) | 150 (86.7) |  |
| HIV status |  |  |  |  |
| Negative | 1,196 (85.4) | 1,071 (87.3) | 125 (72.3) | 0.52 |
| Positive | 27 (1.9) | 23 (1.9) | 4 (2.3) |  |
| Not done/Unknown | 177 (12.6) | 133 (10.8) | 44 (25.4) |  |

Values are in number (%). TB, tuberculosis; IDU, injecting drug user; CXR: chest radiograph; TB-CXR, abnormalities on CXR consistent with tuberculosis; MDR-TB, Multi-drug resistant TB; AFB, acid-fast bacilli; NAA, Nucleic Acid Amplification.
